# Psychometric evaluation of the Family Focused Mental Health Practice Questionnaire in measuring home visitors’ family focused practice

**DOI:** 10.1371/journal.pone.0203901

**Published:** 2018-09-13

**Authors:** Rachel Aine Leonard, Mark Linden, Anne Grant

**Affiliations:** School of Nursing and Midwifery, Medical Biology Centre, Queen’s University, Belfast, Northern Ireland; Hong Kong Polytechnic University, HONG KONG

## Abstract

**Background:**

Worldwide maternal mental illness poses a major public health issue. Supporting maternal mental health and family health is a core aspect of home visiting. Increasingly the benefits of family focused treatments to maternal mental illness are being recognised. However, there are few reliable and valid measures that attempt to assess this type of practice.

**Objectives:**

To explore the psychometric properties of the Family Focused Mental Health Practice Questionnaire in a population of home visitors.

**Methods:**

Home visitors (n = 230) from across a single region of the United Kingdom completed the Family Focused Mental Health Practice Questionnaire. Participants were all females, had a mean age of 44 years, and had an average of 11 years’ experience of home visiting. Exploratory factor analysis was used to explore the factor structure of the questionnaire in this population while Cronbach’s alpha was used to assess the internal consistency of questionnaire subscales.

**Results:**

Exploratory factor analysis revealed a 3-factor solution where each factor contained at least three questionnaire items and had eigenvalues ≥ 1.0. Checks for internal consistency revealed that one factor was unsatisfactory (α < 0.6), which was subsequently discarded. A further exploratory factor analysis supported a 2 factor solution. The factors were named: professional influences on family focused practice and organisational influences on family focused practice. Cronbach’s alpha for the new scale was 0.949.

**Conclusion:**

As home visitors play a key role in supporting parents who have mental illness and their families, it is important to assess their practice using a reliable measure. Our psychometric evaluation has created a more valid, reliable and concise measure that can be used to examine home visitors’ family focused practice.

## Introduction

Maternal mental illness is increasingly recognised as a major public health issue, across high and low income countries [1,2]. Following pregnancy and the postnatal period mothers experience social, physical and mental changes which can increase their vulnerability for the onset or relapse of mental illness [3–5]. Approximately 10% of pregnant women, and 13% of those who have given birth in high-income countries, experience some type of mental disorder, primarily depression or anxiety [5, 6]. There is a growing body of evidence suggesting that maternal mental illness is linked to a wide range of adverse child outcomes [7,8], from birth [9] into adulthood [10]. In addition to adverse child outcomes, the burden of care which can be placed on partners and adult family members, can leave them at increased risk of, emotional distress, stigma, financial burden, lack of support networks, and social exclusion [11,12]. On this basis, there is increasing awareness of the need for early identification and family focused treatment to support mothers who have mental illness and their families [13–15].

Supporting maternal mental health and family health is a core aspect of home visiting [16,17]. In the UK ‘health visitors’ are registered nurses or midwives who have undertaken a specialised degree in public health. The term ‘health visitor’ is mainly used in the UK, Denmark and Norway, however there are comparable professions in Sweden, the US, Canada, Ireland and Finland [18]. They provide a universal service for all families with children aged 0–5 years, offering support for all the family from the ante-natal period until the child begins school. Research to date has suggested that home visitors are in a crucial position to support mother’s mental health, as well as supporting the family as a whole [19]. However, research has also highlighted that home visitors have limited understanding of mental illness [20]. Psychological and psychosocial interventions, such as cognitive behavioural therapy, behavioural activation, or interpersonal therapy, remain the recommended treatment for mental illness during the postnatal period [21], despite poor accessibility to treatment [22] and, little evidence on their effectiveness when delivered by home visitors [23]. While there are some approaches in home visiting that could be classified as family focused, such as the Solihull approach [24], there is limited research to date that has explored their family focused practice (FFP) with mothers who have mental illness and their families.

Family focused practice has been defined as an umbrella term encompassing a continuum of family focused activities, interventions and practices [25–27]. Family focused practices extend across a spectrum of activities, from simply acknowledging the children and partners of service users, to providing in-depth family therapy, such as those which consider the parent, child and the family in a holistic sense [13]. The concept of FFP is underpinned by theories such as ecological theory, and family system theory [28, 29]. Family focused practices have been shown to have beneficial outcomes for mothers, children, and families as a whole [30–32]. Nonetheless, the term can be used interchangeably throughout the literature with terms such as: ‘whole family’, ‘family-oriented’, ‘family-sensitive’ and ‘family-centred’ [26], which has created difficulties in standardising how the term is used and defined [27]. In turn this has created challenges in how the concept can be measured. Additionally, there is currently only one validated scale specifically designed to measure health professionals’ FFP with parents who have mental illness [33].

The Family Focused Mental Health Practice Questionnaire (FFMHPQ) [33] is a tool developed to measure family focused activity, relating to key worker and organisational factors that enable and/or hinder FFP. The original measure was developed in Australia for use with mental health nurses. The FFMHPQ has 16 subscales, comprising a total of 45 items, which are scored on a seven-point Likert scale (ranging from strongly disagree = 1, to strongly agree = 7). Five subscales measure family focused activities such as parenting support, referring family members to services and collaboration with other professionals. The remainder measure organisational factors that can impact these activities such as, workplace support, and workload. Since the development of the FFMHPQ, it has been adapted in a number of studies within Australia [33–35], Ireland [36], Thailand [37], Norway [38,39] and, Northern Ireland [40]. The majority of the studies to date were conducted with a sample of mental health professionals. Principal components analysis of the original scale in an Australian study, revealed a sixteen-factor solution, consisting of forty-five items. However, there was poor internal consistency in three of the sixteen subscales. In addition, some of the remaining factors consisted of two items, indicting they may have been unstable [41]. Despite demonstrably poor internal consistency ratings on the three subscales, some authors have continued to utilise them [33, 36,37,42,43]. Grant [36], administered the FFMHPQ to 343 psychiatric nurses, where poor reliabilities resulted in six of the subscales being excluded from analysis [36]. An adapted version of the FFMHPQ was also used with a sample of preschool teachers and childcare professionals in Australia [35]. Eight of the original sixteen factors were removed based on the research team’s decision that they were not appropriate for teachers. Five of the remaining subscales possessed Cronbach’s alpha values below 0.5, consequently items were removed to improve reliability. Other studies have attempted to adapt the FFMHPQ without testing reliability [37,39]. With the exception of Maybery et al’s [33] Australian research with metal health nurses, no study to date has performed a psychometric evaluation of the FFMHPQ.

Based on previous adaption and validation studies of the FFMHPQ, the aim of the present study is to explore the psychometric properties and factorial structure of the original FFMHPQ in a population of home visitors. To our knowledge this is the first study that has used the FMFHPQ within this population.

## Materials and methods

### Design

A cross-sectional design was utilised to recruit a sample of home visitors from teams across a single region of the United Kingdom. Home visitors were included if they had a minimum of six months post qualifying experience, had direct contact with families, were permanent members of staff (either full time or part time), and possessed adequate understanding of the English language to understand the questionnaire. Home visitors working within the Nurse Family Partnership team, home visitors with no contact with families, agency staff, managerial level staff, student home visitors, community psychiatric nurses and midwives were excluded.

### Participants

At the time of initiation of the study the population consisted of 488 home visitors. Through convenience sampling we obtained a total sample of 230 home visitors. All home visitors were female with a mean age of 44 years. Participant demographics were collected through Part 1 of the distributed questionnaire. See Table 1 for participant characteristics. Part 2 comprised the FFMHPQ.

**Table 1:** Participant demographics.

**Variable**	**Mean**	**SD**
Age	44 (years)	9.29
Length in Practice	11 (years)	9.43
Case Load	223	60.8
FFMHPQ score	210	18.5
**Location of Service**	**N**	**%**
Urban	68	29.8
Rural	73	32
Urban and Rural	86	37.7
**Basis of employment**	**N**	**%**
Full-time	130	57
Part-time	93	40.8
Other	3	1.3
**Currently in a specialist position**	**N**	**%**
Yes	51	22.4
No	174	76.3
**Training in the past 3 years**	**N**	**%**
Yes	210	92.1
None	18	7.9
**Professionals’ experience of Mental health problems?**	**N**	**%**
No experience	92	40.4
Personal Experience	42	18.4
A parent	24	10.5
A sibling	27	11.8
Your child/children	4	1.8
More than one of the above	29	12.7
Missing data	10	4.4
**Parent**	**N**	**%**
Yes	199	87.3
No	29	12.7

### Measure

The FFMHPQ has 16 subscales, comprising a total of 45 items, which are scored on a seven-point Likert scale (ranging from strongly disagree = 1, to strongly agree = 7). Due to poor ratings of internal consistency, three of the subscales were removed for this study. These included: location issues (α = 0.41) which assessed transport and services to refer family members to a particular area; engagement issues (α = 0.42) which examined the opportunity to engage with family members; and support to carers and children (α = 0.58) which referred to the level of information, advocacy and referral provided to carers and children (33). This resulted in 13 subscales, comprising 41 items included in the present study. A low score in a particular subscale suggests a reduced family focus, and a high score an increased family focus. With the potential minimal score being 41 and the highest being 287. The scale required minor adaptation for a UK context and for our population of interest; the term ‘consumer parent’ was changed to ‘service user’ and the term ‘worker’ was changed to ‘home visitor’. In addition to the FFMHPQ, demographic information was also collected. This included: gender; age; time qualified; training received; named healthcare Trust; specialisms; time in current position; location of the team (i.e. rural/urban); parental status; personal experience of mental illness.

### Procedure

Home visitor’s FFP was assessed using the FFMHPQ. The questionnaire was disseminated through staff meetings, which ran approximately every month. At the end of the meeting the researcher was allotted time to introduce the study and give home visitors the opportunity to complete the questionnaire and ask any questions about the research. Participants were then invited to complete the FFMHPQ but were also given the option of taking the questionnaire away, to complete in their own time. If Participants chose to take the questionnaire away, they were provided with a stamped-addressed envelope to return the questionnaire to the research team. Ethical approval was granted by West of Scotland Research Ethics Committee 3 (17/WS/0131) and was conducted in accordance with the Declaration of Helsinki 1975.

### Statistical analysis

As this was the first factor analysis of the FFMHPQ within a population of home visitors, it was deemed that an exploratory approach would be most appropriate. Factor analysis can reveal the underlying factor structure of latent variables through partitioning the shared, unique and error variance of a variable [38]. As FFP is a latent construct, an Exploratory Factor Analysis (EFA) was employed. Data met the underlying assumptions for conducting EFA as shown by Bartletts’s test of sphericity (p < 0.001), and the Keiser-Olkin index (KMO = 0.907). An EFA was performed using principal axis factor extraction and oblique, direct oblimin rotation. Scores of 203 home visitors were included in the EFA, which met a 5:1 ratio of participants to items (n = 41) [41]. As recommended by Kaiser [44], retained factors had eigenvalues greater than 1.0, which was confirmed by the visual inspection of a scree plot. In addition, only factors with minimal cross-loading, those containing at least three items [41] and those with item loadings above .32 [45], were retained. Cronbach’s alpha was used to estimate internal consistency of the retained factors and to determine the best fit for items which cross-loaded. As recommended, factors scoring less than α = 0.6 were discarded [46]. EFA was performed a second time to confirm these results. All statistical analyses were undertaken using STATA version 12.1 software [47].

## Results

Our sample consisted of 230 female home visitors (see Table 1), of whom the mean age was 44 years (SD = 9.29). The mean length in practice was 11 years (SD = 9.43), with the majority in full-time employment (n = 130, 57%). Home visitors worked in rural (n = 73, 32.5%), urban (n = 69, 30.3%) and both rural and urban areas (n = 86, 37.7%). Caseloads ranged from 20 families to 333 families. Seventy-seven percent of home visitors were not in a specialist position, in addition 92% had undertaken training within the last 3 years. The majority of home visitors had received training in perinatal mental health (n = 169, 73.8%) and child-focused training (n = 171, 74.7%). Participants had a total mean FFP score of 210 (SD = 18.5) as measured by the FFMHPQ. The lowest score on this scale was 163 while 260 was the highest.

### Exploratory factor analysis 1

EFA of the FFMHPQ initially revealed a 3-factor solution, consisting of 28 items. Recommended criteria of extracting factors with eigenvalues > 1, items loading above .30, factors with few items cross-loading and containing a minimum of three items [41, 44, 45] were followed while conducting the EFA. One item (no. 24) cross-loaded between 2 factors and was subsequently assigned to the factor with the stronger association. (See Table 2 for the 3-factor solution).

**Table 2:** Factor loading of 28 items across 3 factors.

Item	Factor 1	Factor 2	Factor 3
1. Children and families ultimately benefit if health professionals work together to solve the family’s problems	**0.997**		
2. I am skilled in working with service users in relation to maintaining the wellbeing and resilience of their children	**0.987**		
3. I would like to undertake training in future to increase my skills and knowledge about helping service users with their parenting	**0.986**		
4. I want to have a greater understanding of my profession in a healthcare team approach to working with children and families	**0.981**		
5. I am not able to determine the level of importance that service users place on their children maintaining strong relationships with others outside the family (e.g. other children/peers, school)	**0.977**		
6. There is time to have regular contact with other agencies regarding parents, families or children	**0.974**		
7. I am not confident working with children of service users	**0.971**		
8. I am not experienced in working with child issues associated with parental mental illness	**0.969**		
9. I provide education sessions for adult family members (e.g. about the illness, treatment)	**0.969**		
10. I am knowledgeable about the key things that parents service users could do to maintain the wellbeing (and resilience) of their children	**0.706**		
11. Team-working skills are essential for all health care professionals providing family-focused care	**0.706**		
12. I would like to undertake future training to increase my skills and knowledge for working with children of service users	**0.705**		
13. I regularly provide information (including written materials) about mental health issues to the children of service users	**0.699**		
14. I often consider if referral to parent support programme (or similar) is required by service users	**0.697**		
15. My workplace does not provide supervision and/or mentoring to workers undertaking family focused practices		**0.636**	
16. My workplace provides little support for further training in family focused practices		**0.573**	
17. There are no family therapy or family counselling services to refer service users and their families		**0.558**	
18. There are no parent-related programs (e.g. parenting skills) to refer service users to		**0.456**	
19. I refer service user to parent-related programs (e.g. parenting skills)		**0.448**	
20. I often receive support from co-workers in regard to family focused practice		**0.427**	
21. Government policy regarding family focused practice is very clear		**0.398**	
22. I regularly have family meetings (not therapy) with service users and their families		**0.395**	
23. I am not confident working with families of service user’s		**0.386**	
24. I do not refer children of service user-parents to child focused (e.g. peer support) programs (other than child and adolescent mental health)		**0.346**	0.312
25. I provide written material (e.g. education and information) about parenting to service users		**0.304**	
26. In my workplace other health visitors encourage family focused practice			**0.606**
27. I am able to determine the level of importance that service users place on their children maintaining strong relationships with other family members (e.g. other parent, siblings)			**0.502**
28. I am able to assess the level of children’s involvement in their parent’s symptoms			**0.477**

### Assessment of internal consistency

Cronbach’s alpha for the 3-factor solution identified through EFA was 0.914. Cronbach’s alpha scores for individual factors revealed poor internal consistency for factor 3, which was then removed (See Table 3). The cronbach’s alpha score of factor 2 revealed questionable consistency (α = 0.606). In order to improve consistency any item with factor loading < 0.3 were removed (n = 5). This resulted in an increased alpha value for factor 2 (α = 0.669). The 2-factor solution contained 20 items and possessed good internal consistency between items. Cronbach’s alpha for the new 20 item FFMHPQ was 0.949.

**Table 3:** Cronbach’s alpha scores for the 3-factor solution.

Factor	α
1	0.972
2	0.669
3	0.516*

*denotes factors which were removed due to **α** < 0.6

### Exploratory factor analysis 2

A further EFA was preformed to confirm the results of the 2-factor solution. EFA revealed a 2-factor solution with 20 items. Factor loading scores supported our conclusion of the best fit for the 20 items within the 2 factors (see Table 4). Examination of the scree plot supported this conclusion, showing the point of inflection at the 2^nd^ factor. The factors were named as: professional influences on FFP and organisational influences on family focused practice.

**Table 4:** Factor loading for 20 items across 2 factors.

Item	Factor 1	Factor 2
Children and families ultimately benefit if health professionals work together to solve the family’s problems	**0.997**	
I am skilled in working with service users in relation to maintaining the wellbeing and resilience of their children	**0.987**	
I would like to undertake training in future to increase my skills and knowledge about helping service users with their parenting	**0.986**	
I want to have a greater understanding of my profession in a healthcare team approach to working with children and families	**0.981**	
I am not able to determine the level of importance that service users place on their children maintaining strong relationships with others outside the family (e.g. other children/peers, school)	**0.977**	
There is time to have regular contact with other agencies regarding parents, families or children	**0.974**	
I am not confident working with children of service users	**0.971**	
I am not experienced in working with child issues associated with parental mental illness	**0.969**	
I provide education sessions for adult family members (e.g. about the illness, treatment)	**0.969**	
I am knowledgeable about the key things that parents service users could do to maintain the wellbeing (and resilience) of their children	**0.706**	
Team-working skills are essential for all health care professionals providing family-focused care	**0.706**	
I would like to undertake future training to increase my skills and knowledge for working with children of service users	**0.705**	
I regularly provide information (including written materials) about mental health issues to the children of service users	**0.699**	
I often consider if referral to parent support programme (or similar) is required by service users	**0.697**	
My workplace does not provide supervision and/or mentoring to workers undertaking family focused practices		**0.636**
My workplace provides little support for further training in family focused practices		**0.573**
There are no family therapy or family counselling services to refer service users and their families		**0.558**
There are no parent-related programs (e.g. parenting skills) to refer service users to		**0.456**
I refer service user to parent-related programs (e.g. parenting skills)		**0.448**
I often receive support from co-workers in regard to family focused practice		**0.427**

## Discussion

The objective of the present study was to investigate the psychometric properties of the FFMHPQ for assessing home visitors’ FFP with mothers who have mental illness and their families. Our analysis suggested a 2-factor solution in our population of home visitors, with 20 items from the original FFMHPQ included, and 21 items removed. The internal consistency of this newly proposed 20-item version, was found to be excellent with a Cronbach’s alpha of 0.949.

Since the development of the original scale, there have been several attempts to adapt the FFMHPQ. Some studies have adapted the FFMHPQ without attempting to validate their changes [37,39], while those that tested internal reliabilities revealed many of these to be unacceptable [35,42]. To our knowledge this is the only study since the development of the original FFMHPQ that has attempted to explore the factorial structure of the FMFHPQ. The EFA performed in this study revealed a different structure than the original FFMHPQ, which suggested a sixteen-factor solution with 45 items, compared with 2 factors and 20 items in the present study. It is important to note that the variability in factor solutions and internal reliability of previous versions may be due to differences between samples. The majority of previous research [37,40,48] has consisted of mental health professionals, while the present study comprised home visitors. While the two professions may have underlying similarities, mental health professionals will have specialised mental health training, whereas home visitors often receive minimal training in mental health, and thus cannot be compared. The second explanation may be due to some limitations in the original principal components analysis which was performed by Maybery et al [33]. For example, the original study opted to conduct a principal component analysis instead of a factor analysis, which is the preferable method when considering latent variables [41]. Secondly, items were divided into three factors prior to the principal components analysis, which is not common practice. Finally, some factors were retained in the final version despite having fewer than three items or had poor reliabilities (< 0.5), which is contrary to accepted practice [41,45].

Even with the highlighted limitations, the FFMHPQ provides a much needed means of measuring the concept of FFP. Family-focused practice recognises a person’s needs within the context in which they live, identifying both the individuals’ needs and those of the family as a whole [49]. This approach can promote parents’ recovery [50, 7], reduce the risk of transmission of mental illness to children [31,51], and reduce the burden placed on carers and partners [12,52]. While the benefits of FFP have been acknowledged, its adoption into practice is not without its challenges. Barriers to effective FFP include; staffing shortages, lack of staff training, professional attitudes towards FFP, issues of family engagement, and organisational culture [53–55]. Maybery et al. [56] found that professional skill and knowledge of FFP were key enablers to effective FFP. In addition, availability of services, co-worker support, and assessing connectedness between parent and child have also been identified as important enablers [43,48]. The majority of the enablers and barriers identified have been from studies exploring mental health professionals’ practice, with few studies exploring home visiting practices.

There is consensus in the FFP literature to date, that there are challenges in how the concept of FFP is defined and used within practice and research [28,29,53]. In turn this has created challenges in how FFP can be measured. To assess the extent of professionals’ FFP, there needs to be a means of measurement which is reliable and valid. Through assessment of practice we can identify areas of effective practice and areas for improvement. Our psychometric evaluation has created a more valid, reliable and concise questionnaire which was successfully employed to test FFP among home visitors.

There are certain characteristics within our sample that may have influenced the variability noticed in scores on the FFMHPQ. Home visitors had caseloads ranging from 20 to 333 families. Those with fewer cases were more likely to be in specialist positions and were consequently more likely to score highly on the FFMHPQ. It is widely acknowledged that home visitors’ caseloads are increasing, as is the complexity of their cases [57]. While there are no national guidelines on optimum caseload numbers in the UK, the Community Practitioners and Health Visitors Association (CPHVA), a UK organisation, recommend an average caseload of 250 for safe and effective practice [58]. Internationally, the challenges of time and increasing caseloads are echoed in Australia [59], however, in Denmark the maternal and child health service is legislated to allocate each home visitor a maximum of 150 children [60]. Increasing caseloads of greater complexity result in a lack of time spent with families which has been shown to be a significant barrier to effective FFP [43]. Personal experiences of the home visitor can also shape their identity as a professional [61]. Some studies within mental health nursing have shown that personal experience of mental illness can have a positive influence on their understanding and relationship with service users [62, 63]. Over half of our sample (n = 127, 59.8%) had personal experience of mental illness, however, little is known about how much influence this factor has on home visiting practice.

Distribution of home visitors between urban, rural and urban & rural areas was evenly split in our sample. Although, urban regions are normally associated with areas of poorer health and deprivation [64, 65], families in rural regions have poorer access to services, including home visiting [66]. Home visitors based in rural areas oversee cases in widely distributed geographic locations, travel between which may stretch their already limited time with families. Referral to services is an important component of FFP working, however, home visitors in rural areas have less access to services which impacts on their ability to adequately support their clients. Other important aspects of FFP include, training, knowledge and skill development [43], which have been shown to improve confidence when working with families. Within our sample, only 9.2% of home visitors had received any family focused training which may have adversely impacted their confidence.

This study is not without its own limitations. While a 5:1 ratio of participants to items is deemed adequate, a 20:1 would have been ideal [41]. Although this would have required the authors to obtain a sample of 820 participants, which would have exceeded the total population of home visitors available. In addition, due to the cross-sectional nature of our study design, it was not possible to perform test re-test reliability. In light of these limitations, future studies are needed which employ rigorous psychometric procedures to further assess the validity and reliability of the FFMHPQ with new samples of home visitors in the UK and internationally.

## Conclusion

Being family focused is a central aspect in supporting vulnerable families. If practice is to be improved, it is important that we possess a reliable means to assess performance. By assessing the validity and reliability of the FFMHPQ we have improved upon a tool to assess FFP among practitioners who have the potential to influence the recovery of vulnerable women with mental illness and their families. Evaluation of FFP can inform decisions about improving service delivery. However, this evaluation should be based on the best available evidence, using valid and reliable measures. The ability to assess the performance of home visiting professionals, would facilitate targeted training to improve practice and explore the impact of key variables, such as caseload, experience, and resources on FFP. If we are to improve the quality of evidence, future studies are needed which employ rigorous psychometric procedures to further assess the validity and reliability of the FFMHPQ with new samples of home visitors in the UK and internationally.
